# Functional alterations in large-scale resting-state networks of amyotrophic lateral sclerosis: A multi-site study across Canada and the United States

**DOI:** 10.1371/journal.pone.0269154

**Published:** 2022-06-16

**Authors:** Komal Bharti, Simon J. Graham, Michael Benatar, Hannah Briemberg, Sneha Chenji, Nicolas Dupré, Annie Dionne, Richard Frayne, Angela Genge, Lawrence Korngut, Collin Luk, Lorne Zinman, Sanjay Kalra, for the Canadian ALS Neuroimaging Consortium (CALSNIC)

**Affiliations:** 1 University of Alberta, Edmonton, Alberta, Canada; 2 University of Toronto, Toronto, Ontario, Canada; 3 University of Miami, Miami, Florida, United States of America; 4 University of British Columbia, Vancouver, British Columbia, Canada; 5 University of Calgary, Calgary, Alberta, Canada; 6 Université Laval, Quebec, Quebec, Canada; 7 McGill University, Montreal, Quebec, Canada; University of Naples Federico II, ITALY

## Abstract

Amyotrophic lateral sclerosis (ALS) is a multisystem neurodegenerative disorder characterized by progressive degeneration of upper motor neurons and lower motor neurons, and frontotemporal regions resulting in impaired bulbar, limb, and cognitive function. Magnetic resonance imaging studies have reported cortical and subcortical brain involvement in the pathophysiology of ALS. The present study investigates the functional integrity of resting-state networks (RSNs) and their importance in ALS. Intra- and inter-network resting-state functional connectivity (Rs-FC) was examined using an independent component analysis approach in a large multi-center cohort. A total of 235 subjects (120 ALS patients; 115 healthy controls (HC) were recruited across North America through the Canadian ALS Neuroimaging Consortium (CALSNIC). Intra-network and inter-network Rs-FC was evaluated by the FSL-MELODIC and FSLNets software packages. As compared to HC, ALS patients displayed higher intra-network Rs-FC in the sensorimotor, default mode, right and left fronto-parietal, and orbitofrontal RSNs, and in previously undescribed networks including auditory, dorsal attention, basal ganglia, medial temporal, ventral streams, and cerebellum which negatively correlated with disease severity. Furthermore, ALS patients displayed higher inter-network Rs-FC between the orbitofrontal and basal ganglia RSNs which negatively correlated with cognitive impairment. In summary, in ALS there is an increase in intra- and inter-network functional connectivity of RSNs underpinning both motor and cognitive impairment. Moreover, the large multi-center CALSNIC dataset permitted the exploration of RSNs in unprecedented detail, revealing previously undescribed network involvement in ALS.

## Introduction

Amyotrophic lateral sclerosis (ALS) is a neurodegenerative disorder characterized by progressive degeneration of both upper motor neurons (UMN) and lower motor neurons [1–3]. ALS affects a wide range of neuronal networks engaged in motor regulation [3]. Extra-motor brain regions are also directly compromised in ALS, and may result in abnormal cognitive and behavioural function [4–6]. Changes in brain function can be investigated in detail using resting-state functional magnetic resonance imaging (Rs-FMRI) [7–9]. Rs-FMRI is a commonly used imaging technique to capture synchronous low-frequency fluctuations in blood oxygen level-dependent (BOLD) signals between functionally connected brain regions [10] in the absence of stimulus-driven tasks [11], and thus can help detect abnormal functioning in pathological conditions where task-based imaging is impractical or logistically challenging.

Previous Rs-FMRI studies have demonstrated the role and importance of several seed-based correlation maps i.e. RSNs in ALS pathophysiology [7, 9, 12–21]. Some of these studies have used an *a-priori* approach to examine functional alterations in the RSNs associated with the cortical and subcortical brain areas such as the motor cortex, frontal cortex, cerebellum and basal ganglia corresponding to the motor as well as cognitive decline in ALS [7–9, 12, 14, 22, 23]. Alternatively, some Rs-FMRI studies have used a data-driven, independent component analysis (ICA) approach which elucidates whole-brain RSNs without predefined seed regions [13, 15, 17–19, 21]. In ALS, such studies have mainly reported sensorimotor RSN, default mode RSN, and fronto-parietal RSN [13, 16, 18, 21] suggesting the reorganization of motor and extra-motor domains of functional brain networks in of ALS patients. The technique avoids the probability of missing connectivity differences due to seed selection [24–27]. In addition, ICA helps to examine multiple RSNs and thus provides more global information [28, 29] and the potential to probe underlying mechanisms. Keeping in light both approaches, a widespread involvement of not only motor areas, but also extra-motor brain areas actively involved in the underlying pathophysiology of ALS were revealed suggesting not just functional impairment of one or more RSNs, but rather a result of failed communication or interplay of multiple RSNs across the whole brain.

Considering the fact that abnormal whole brain resting state functional connectivity (Rs-FC) plays an important role in the pathophysiology of ALS, we sought to examine whole brain within-network and between-network Rs-FC in a large cohort of ALS patients from multiple centres in Canada and the United States. In addition, we also wished to explore the association of intra-network and inter-network Rs-FC with clinical features relevant to motor and extra-motor dysfunction in ALS patients.

## Methods

### Canadian ALS Neuroimaging Consortium

Data were acquired for the study across North America through the Canadian ALS Neuroimaging Consortium (CALSNIC) [30]. The main objective of CALSNIC is to develop novel MRI biomarkers for ALS. There are 2 projects in CALSNIC: CALSNIC 1 and CALSNIC 2. The 5 sites of data acquisition for CALSNIC 1 were: University of Alberta, *Edmonton*; University of Calgary, *Calgary*; McGill University, *Montreal*; University of Toronto, *Toronto*; and University of British Columbia, *Vancouver*. For CALSNIC 2, data were acquired from the aforementioned sites except Vancouver; and additionally, from University of Miami, *Miami*, and University of Laval, *Quebec*.

### Participants

A total cohort of 235 participants (115 HC; 120 ALS patients) were recruited through CALSNIC. Patients were diagnosed as possible (25), probable (60), or definite ALS (35) according to revised El Escorial Criteria [31]. Out of 120 ALS patients, 20 patients had bulbar onset and 100 patients had limb onset. The exclusion criteria included the presence of other neurological and psychiatric illness. Excluded were patients with primary lateral sclerosis, progressive muscular atrophy, and frontotemporal dementia. For the control group, 115 age and sex matched HC were recruited with no history of neurological and psychiatric illness. The study was conducted with the approval of the Health Research Ethics Board of each participating site and informed written consent was obtained from the participants.

### Clinical assessment

All patients underwent a neurological exam administered by a trained neurologist at each participating site. The ALS functional rating scale revised (ALSFRS-R) was used as a measure of disease severity [32]. Decreased ALSFRS-R corresponds to greater clinical disability. A disease progress rate was subsequently calculated using the ALSFRS-R scores and symptom duration [33]. Severity of upper motor neuron (UMN) degeneration in the limbs and bulbar regions was inferred for patients by calculating an UMN burden score derived from the neurological exam. A UMN burden score was calculated with a maximum score of 12. The presence of spasticity and hyperreflexia in the upper and lower limbs, Babinski sign, and clonus at each ankle were tabulated with a maximum possible score of 6 from each side of the body [34]. Further assessment of motor impairment was measured using finger tapping and foot tapping tasks for the right side and the left side. The Edinburgh Cognitive and Behavioral ALS screen (ECAS) was used to test cognitive performance [35]. In addition to the ECAS "Total" score, the "ALS Specific" and "ALS Non-specific" were also noted. The ECAS and tapping tasks were also performed by each participant in the healthy control group.

### Imaging protocol

Structural and functional images were acquired using 3 Tesla MRI systems at all sites. Prior to Rs-FMRI, participants were instructed to remain still, awake, and with their eyes closed, in a fully relaxed condition. The details of data acquisition across the different sites are summarized in Table 1 [30].

**Table 1:** Details of MRI data acquisition across research centres in Canada and United States.

Project	*CALSNIC-1*					*CALSNIC-2*					
Site	*University of Alberta*	*University of British Columbia*	*University of Calgary*	*McGill University*	*University of Toronto*	*University of Alberta*	*University of Calgary*	*McGill University*	*University of Toronto*	*University of Miami*	*Universite Laval*
Scanner model	Siemens 3T Prisma	Philips 3T Intera	GE 3T Discovery MR 750	Siemens 3T Triotrim	GE 3T Discovery MR 750	Siemens 3T Prisma	GE 3T Discovery MR 750	Siemens 3T Prisma	Siemens 3T Prisma	Siemens 3T Triotrim	Philips 3T Achieva
Software version	syngo MR E11	3.2.31	DV25.0_EB_1442.a	syngo MR B17	DV24.0_R01_1344.a	syngo MR E11	DV25.0_R02_1549.b	syngo MR E11	syngo MR E11	syngo MR B17	5.3.0.3
**3D T**_**1**_ **-weighted scan, axial acquisition**											
*Acquisition orientation*	axial	axial	axial	axial	axial	sagittal	sagittal	sagittal	sagittal	sagittal	sagittal
*Repetition time*, *ms*	2300	7.9	7.4	2300	7.4	1700	8.1	1700	1700	1800	7.1
*Echo time*, *ms*	3.43	3.5	3.1	3.43	3.1	2.21	3.2	2.21	2.21	2.13	3.4
*Inversion time*, *ms*	900	950	400	900	400	880	400	880	880	900	950
*Flip angle*, *degrees*	9	8	11	9	11	10	16	10	10	10	10
*Field of view*	256	240	256	256	256	256	256	256	256	256	256
*Matrix dimension*, *pixels*	256 x 256	240 x 240	256 x 256	256 x 256	256 x 256	232 x 256	256 x 256	232 x 256	232 x 256	256 x 256	256 x 256
*Voxel dimension*, *mm*	1 x 1 x 1	1 x 1 x 1	1 x 1 x 1	1 x 1 x 1	1 x 1 x 1	1 x 1 x 1	1 x 1 x 1	1 x 1 x 1	1 x 1 x 1	1 x 1 x 1	1 x 1 x 1
*Slices*, *n*	176	150	176	176	176	176	176	176	176	176	176
*Acquisition times*, *min*	05:30		4:30	05:30	4:30	03:37	04:16	03:37	03:37	04:10	04:08
**Resting-state functional MRI**											
*Repetition time*, *ms*	2200	2200	2200	2200	2200	2200	2200	2200	2200	2200	2200
*Echo time*, *ms*	30.0	30.0	30.0	30.0	30.0	30	30	30	30	30	30
*Flip angle*, *degrees*	70	70	70	70	70	70	70	70	70	70	70
*Field of view*	224	224	224	224	224	224	224	224	224	224	224
*Matrix dimension*, *pixels*	64 x 64	64 x 64	64 x 64	64 x 64	64 x 64	64 x 64	64 x 64	64 x 64	64 x 64	64 x 64	64 x 64
*Voxel dimension*, *mm*	3.5 x 3.5 x 3.5	3.5 x 3.5 x 3.5	3.5 x 3.5 x 3.5	3.5 x 3.5 x 3.5	3.5 x 3.5 x 3.5	3.5 x 3.5 x 3.5	3.5 x 3.5 x 3.5	3.5 x 3.5 x 3.5	3.5 x 3.5 x 3.5	3.5 x 3.5 x 3.5	3.5 x 3.5 x 3.5
*Trains*, *n*	192	192	192	192	192	250	250	250	250	250	250
*Slices*, *n*	40	40	40	40	40	40	40	40	40	40	40
*Acquisition times*, *min*	07:11		~7:00	07:11	~7:00	09:18	09:10	09:18	09:18	09:17	09:23

### Data analysis

Structural and functional data analysis were performed using the FSL (Functional Magnetic Resonance Imaging of the Brain [FMRIB] Software library packages) (http://www.fsl.fmrib.ox.uk/fsl/fslwiki) [36].

**Fig 1:**
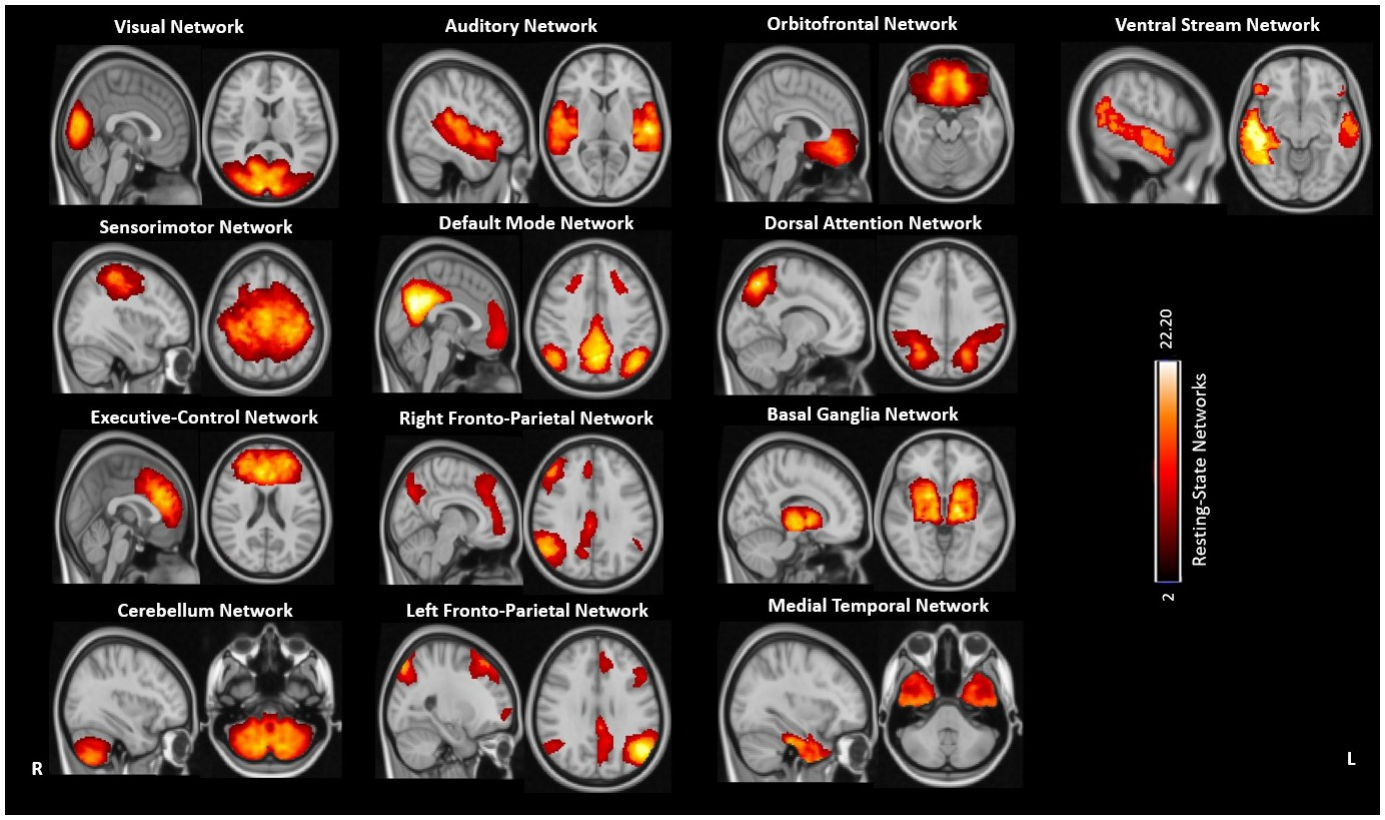
Resting state networks (RSNs) extracted from the whole group of participants using the Melodic—Group ICA approach. The color bar represents the range of intensity voxels. Brain maps are shown in radiological convention.

Preprocessing steps included data analysis of all the subjects using FSL fMRI expert analysis tool (FEAT, http://www.fsl.fmrib.ox.uk/fsl/fslwiki). Brain extraction was performed on the three dimensional (3D) T1-weighted images using the brain extraction tool (BET) [37]. The Rs-FMRI data were corrected for head motion using the MCFLIRT tool, as well as slice timing correction and spatial smoothing using a Gaussian kernel with a full width at half maximum of 5 mm [38]. The data was then subjected to single-session independent component analysis-based automatic removal of motion artifacts (ICA_AROMA) [39] in order to identify independent components (ICs) representing motion-related artifacts. The FMRIB linear image registration tool (FLIRT) (http://www.fmrib.ox.ac.uk/fsl) was used to register the clean fMRI data from motion related IC’s of each participant with the processed BET images. The output of linear registration was then non-linearly registered with the Montreal Neurological Institute (MNI) standard space using FMRIB’s non-linear image registration tool (FNIRT) (http://www.fmrib.ox.ac.uk/fsl). To perform higher level group comparisons using the FSL MELODIC software, a temporal concatenation of spatial ICA maps was performed using temporal high pass filter of 100 seconds [40]. Analysis included variance normalization of time courses and an automatic dimensionality estimation using the temporally concatenated ICA technique. For the post stats, ICA maps were thresholded with mean high-resolution 3D T1 weighted images. The group MELODIC output of 20 components generated automatically from 235 subjects was carefully inspected by an experienced researcher (K.B.) The 13 independent components representing the best RSNs were selected according to the previous literature [41], whereas the other 7 components indicating noise were discarded [27]. Subsequently, the 13 selected RSNs listed in the Fig 1 underwent dual regression analysis. This procedure first regressed the group ICA maps onto each participant’s four-dimensional (4D) dataset to give a set of time-courses; and secondly regressed the time-courses into the same 4D dataset to generate participant-specific spatial maps [29]. These participant-specific spatial maps were then considered for intra- and inter- network Rs-FC analysis.

### Motion parameter analysis

The Rs-FC data are susceptible to head motion artifacts, potentially confounding the assessment of population differences [42, 43]. Absolute (referenced to the middle time-point) and relative (compared to previous time point) values were recorded from the BOLD time series as the root-mean-square values of three translational axis of *X*, *Y*, and *Z*, and rotational axis of pitch, roll, and yaw. After a careful inspection of the recorded motion correction estimates using FSL MCFLIRT tool, subjects with more than 1.5 mm maximum displacement in any of the axis or 1.5° of the angular rotation were excluded from the study [44]. Additionally, any participants with < - 4 minutes of uncorrupted data were excluded from the study [45]. Lastly, the mean absolute, and relative head motion displacement values within 1.5 mm were further subjected for group comparisons using a two-sample t test through SPSS software (IBM, Armonk, New York, USA).

### Intra-network resting state functional connectivity

Intra-network Rs-FC differences were tested by entering participant-specific spatial maps representing the 13 RSNs into a multiple linear regression model implemented in FSL [46]. Age, sex, motion parameters, data acquisition site and MRI system type were included as covariates of no interest in the statistical model. For every participant-specific spatial map representing one of the 13 RSNs (Fig 1), the respective mask was extracted using the ‘fslmath’ command in FSL. The intra-network Rs-FC was then assessed within the mask of the individual RSN using the threshold free cluster enhancement (TFCE) technique in FSL and 5000 nonparametric random permutations [46]. Significant differences in the intra-network Rs-FC were corrected for multiple comparisons using the family wise error (FWE) approach at a significance level of p<0.05. The brain areas with statistically significant findings were extracted using the Harvard-Oxford atlas inbuilt in FSL [47].

### Correlation analysis

In ALS patients, correlation analyses were performed between the intra-network Rs-FC and clinical variables: ALSFRS-R, disease progression rate, symptom duration, finger and foot tapping scores (right and left sides), UMN burden score, ECAS Total, ECAS ALS Specific and ECAS ALS Non-specific. Statistical differences were assessed within the mask of respective RSNs using the TFCE technique involving 5000 nonparametric random permutations, with age, sex, motion parameters, data acquisition site included as covariates of no interest. Moreover, MRI system type was also considered as an additional regressor in the statistical model [48, 49]. Correlations results were corrected for multiple comparisons using the FWE approach at a significance level of p<0.05. The brain areas with statistically significant findings were extracted using the Harvard-Oxford atlas inbuilt in FSL [47].

### Inter-network resting state functional connectivity

Participant-specific spatial maps representing the 13 RSNs were used to investigate inter-network Rs-FC differences using the FSLNets Matlab toolbox (https://fsl.fmrib.ox.ac.uk/fsl/fslwiki/FSLNets). To obtain full and partial correlation matrices, the time series for the 13 RSNs were extracted, normalized and then subjected to correlation analysis. Between-network group comparisons of the normalized time series correlations were performed using a two-sample unpaired t-test with the same covariates of no interest as described above.

### Correlation analysis

Potential associations between inter-network Rs-FC and clinical variables were assessed using Spearman rank-order correlation in SPSS software. The assessment was performed while regressing out the same covariates of interest and with the same threshold for statistical significance as described above.

## Results

### Demographic and clinical scores

Demographic and clinic details of the ALS patients and HC are shown in Table 2. No significant differences were observed in sex between ALS patients and HC (p>0.05). However, age differences were observed to be statistically significant between ALS patients and HC (p<0.05). ALS patients also had lower ECAS scores (Total, Specific and Non-specific) and tapping task scores than the HC group (p<0.05).

**Table 2:** Demographic and clinical features of ALS patients and healthy subjects.

Variables	ALS patients (n = 120)	Healthy Controls (n = 115)	p value
Age (years)	59.3±11.2	55.2±9.7	0.002*
Male/Female	78/52	60/59	0.12
ALSFRS-R	37.5±6.4	--	--
Symptom Duration (months)	33.2±26.2(20–92)	--	--
ECAS-Total	106.2±15.3	112.3±12.4	0.0004*
*ECAS ALS* Specific	78.7±13.1	83.6±10.0	0.0006*
*ECAS ALS* Non-Specific	27.5±3.7	28.7±3.8	0.01*
Right Finger Tapping (10 s)	36.8±20.8	55.5±20.9	0.00001*
Left Finger Tapping (10 s)	33.5±18.6	49.6±19.0	0.00001*
Right Foot Tapping (10 s)	23.4±16.0	39.6±14.3	0.00001*
Left Foot Tapping (10 s)	21.6±15.5	59.6±31.4	0.00001*
UMN Score	5.0±3.5	--	--

*ALS*: Amyotrophic Lateral Sclerosis; ALSFRS-R: Amyotrophic Lateral Sclerosis Functional Rating Scale Revised; ECAS: Edinburgh Cognitive and Behavioural ALS Screen: UMN: Upper Motor Neuron. Values are reported as mean ± SD and (lower bound, upper bound).

Values are reported as mean ± SD (range).

Differences in the demographic and clinical scores between ALS patients and Healthy Controls were assessed by the unpaired t test.

The sex difference between ALS patients and Healthy Controls was assessed by the *χ*^2^ test.

### Motion parameter

The mean absolute head displacement values were 0.32±0.22 mm in ALS patients and 0.28±0.19 mm in HC. The mean relative head displacement values were 0.11±0.08 mm in ALS patients and 0.10±0.05 mm in HC. There were no significant differences in motion parameters between the ALS patients and HC (p>0.05).

### Intra-network resting state functional connectivity

**Fig 2:**
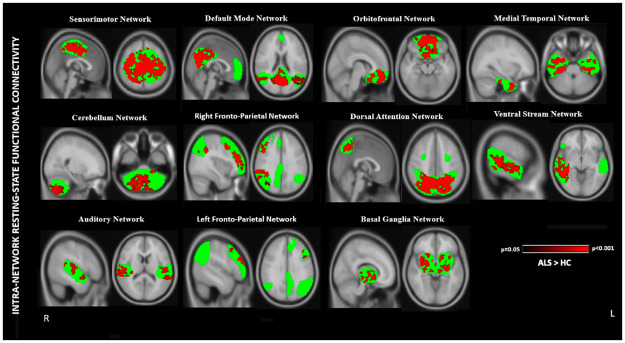
Resting-state networks (RSNs) displayed significant intra-network resting-state functional connectivity (Rs-FC) differences in Amyotrophic lateral sclerosis (ALS) patients compared to healthy subjects (HC). Red: Higher Rs-FC in sensorimotor RSN, cerebellum RSN, auditory RSN, default mode RSN, right fronto-parietal RSN, left fronto-parietal RSN, orbitofrontal RSN, dorsal attention RSN, basal ganglia RSN, medial temporal RSN, ventral stream RSN. Green: Mask obtained from the respective RSNs. Results were corrected for multiple comparisons using the family-wise error approach ate p<0.05. The color bar represents 1-p values. Brain maps are shown in radiological convention.

Compared to HC, ALS patients displayed higher intra-network Rs-FC in 11 RSNs: sensorimotor, cerebellum, auditory, default mode, right and left fronto-parietal, orbitofrontal, dorsal attention, basal ganglia, medial temporal and ventral stream (Fig 2; Table 3) [FWE; p < 0.05].

**Table 3:** Brain areas exhibiting higher intra-network resting-state functional connectivity (Rs-FC) in resting-state networks (RSNs).

Group differences	Resting-state networks	Brain areas	Number of clusters	Clusters voxels	Coordinates			Peak—z stat
					X	Y	Z	
**ALS>HC**								
	**Sensorimotor Network**	**Bilateral**: Anterior and posterior cingulate gyrus, pre and post central gyrus, precuneus, supplementary motor area, superior frontal gyrus, supramarginal gyrus, superior parietal lobule	21	5588	29	67	60	4.7
	**Cerebellum Network**	**Bilateral**: VI, VII a and b, VIII a and b, IX, X, crus I, vermis VIII a **Right:** Crus II **Left:** I-IV	21 20	1716 133	49 56	36 47	19 13	3.7 4.5
	**Auditory Network**	**Bilateral:** Superior and middle temporal gyrus, insula, planum temporale, planum polare, parietal operculum cortex, heschl’s gyrus	46 45 44 43	283 276 115 104	11 69 78 20	44 50 39 63	43 45 36 39	3.4 3.7 3.9 3.6
	**Default Mode Network**	**Bilateral:** Precuneus cortex, cuneal cortex, intracalcarine cortex, supracalcarine cortex, posterior cingulate gyrus, angular gyrus, superior and inferior lateral occipital cortex, posterior supramarginal gyrus, middle temporal gyrus	20 19 18	2316 287 231	40 17 63	31 34 32	39 59 58	4.5 4.6 3.5
	**Right Fronto-Parietal Network**	**Right:** Superior, middle, and inferior frontal gyrus, precentral gyrus, superior parietal lobule, supramarginal gyrus, angular gyrus, lateral occipital cortex	38 37	2457 393	22 22	78 52	31 58	4.0 3.7
	**Left Fronto-Parietal Network**	**Left:** Superior, middle, and inferior frontal gyrus, precentral gyrus	36 33	222 726	29 60	58 79	35 48	3.9 4.3
	**Orbitofrontal Network**	**Bilateral:** Frontal pole, frontal orbital and medial cortex, paracingulate gyrus, subcallosal cortex	32 24	384 3775	73 44	65 62	39 27	4.0 4.9
	**Dorsal Attention Network**	**Bilateral**: Lateral occipital cortex, angular gyrus, precuneus cortex, cuneal cortex, supracalcarine cortex, occipital pole	17	5652	25	26	55	4.7
	**Basal Ganglia Network**	**Bilateral:** Thalamus, putamen, pallidum, and insular cortex, precuneus, posterior cingulate gyrus	27 26	1605 290	43 64	51 57	39 38	4.8 3.9
	**Medial Temporal Network**	**Bilateral:** Temporal fusiform gyrus, parahippocampal gyrus, temporal pole, inferior temporal gyrus, middle temporal gyrus, temporal occipital fusiform cortex	18 17	1451 845	29 65	71 67	15 16	4.0 4.4
	**Ventral Stream Network**	**Right:** Heschl’s gyrus, planum temporale, superior, middle and inferior temporal gyrus, temporooccipital part, posterior supramarginal gyrus, superior and inferior lateral occipital cortex, angular gyrus	18 17	1495 659	30 28	43 26	34 45	4.0 3.9

ALS > HC represents higher whole brain intra-network resting-state functional connectivity in Amyotrophic lateral sclerosis (ALS) as compared to healthy controls (HC). Results were corrected for family-wise error at p < 0.05 (FWE corrected; p < 0.05).

* Number of clusters represents the exact number of clusters in the fMRI analysis stayed significant after correcting the results for multiple comparisons (FWE corrected; p < 0.05).

*Cluster voxel represents number of voxels in each significant cluster.

*Peak z-stat denotes the maximum statistical value (z-stat) for the peak activity

*Coordinates were extracted from MNI 152 space

### Clinical correlations

**Fig 3:**
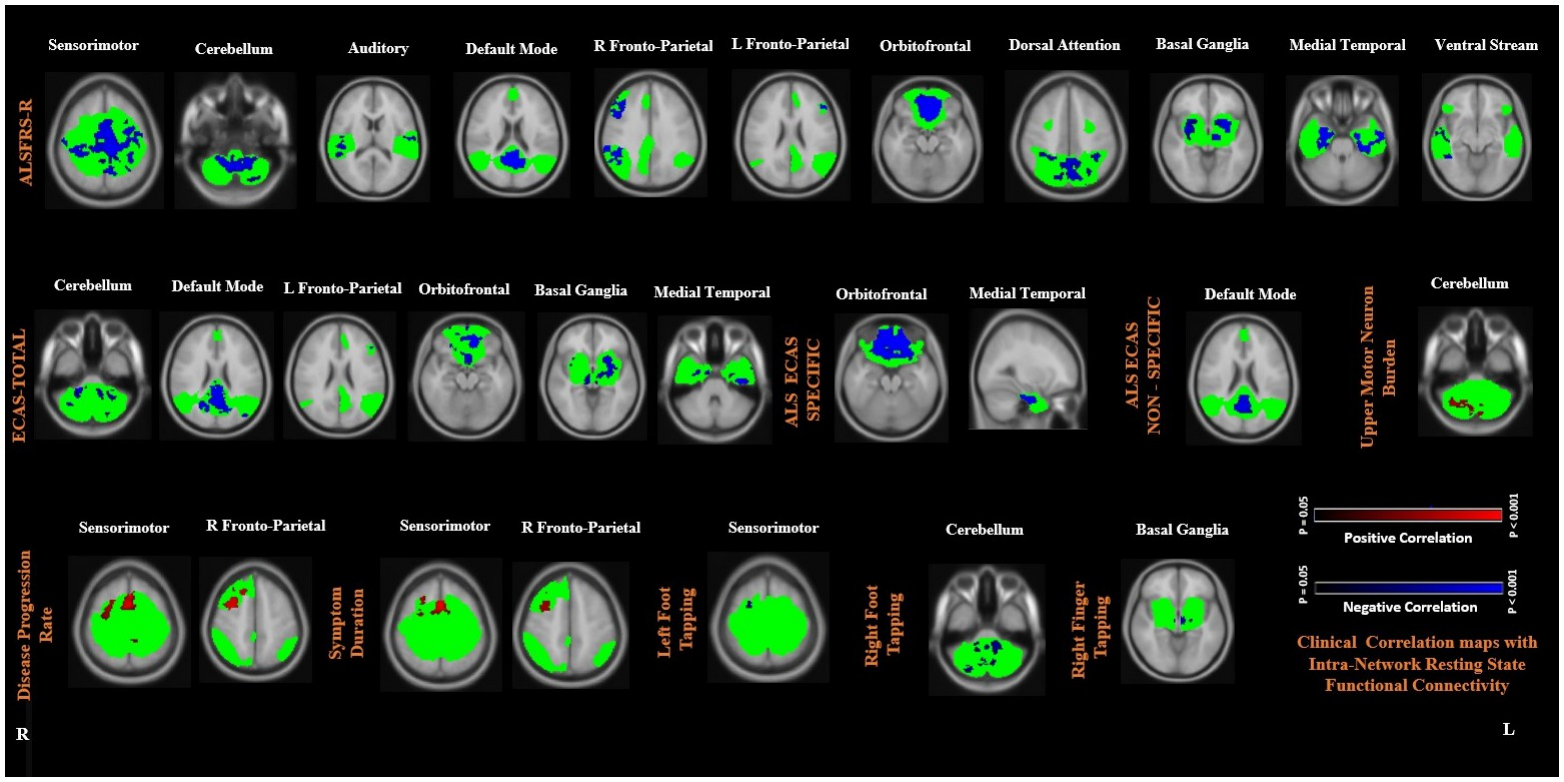
Significant correlations between higher intra-network resting-state functional connectivity (Rs-FC) and the clinical variables of Amyotrophic lateral sclerosis (ALS) patients. ALS functional rating scale revised (ALSFRS-R); Edinburg Cognitive and Behavioral ALS screening (ECAS) Total score and Specific and Non-Specific subdomain scores; Upper motor neuron (UMN) burden; Disease Progression Rate; Symptom duration; Left and Right foot tapping; and Right finger tapping. Red: Positive correlation. Blue: Negative correlation. Green: Mask obtained from the respective RSNs. Results were corrected for the multiple comparisons using the family-wise error approach at p<0.05. Color bar represents 1-p values. Brain maps are shown in radiological convention.

In ALS, intra-network Rs-FC in the sensorimotor, cerebellum, auditory, default mode, right and left fronto-parietal, orbitofrontal, dorsal attention, basal ganglia, medial temporal, and ventral stream RSNs was negatively correlated with ALSFRS-R. Intra-network Rs-FC in the cerebellum, default mode, left fronto-parietal, basal ganglia, orbitofrontal, and medial temporal RSNs was also negatively correlated with ECAS Total score and its sub scores. Conversely, intra-network Rs-FC in the cerebellum RSN was positively correlated with UMN burden scores, and sensorimotor and right fronto-parietal RSNs were positively correlated with the disease progression rate and symptom duration. Lastly, intra-network Rs-FC within sensorimotor, basal ganglia, and cerebellum RSNs negatively correlated with finger and foot tapping scores. Each of these associations is depicted in Fig 3 and summarized in Table 4 (FWE; p < 0.05).

**Table 4:** Brain areas showing positive and negative correlations between higher intra-network resting-state functional connectivity (Rs-FC) in resting-state networks (RSNs) and clinical data.

Clinical parameters and nature of correlation	Resting-state networks	Brain areas	No of clusters	Clusters voxels	Coordinates			Peak–z stat
					X	Y	Z	
**ALSFRS-R *[Negative correlation]***								
	**Sensorimotor Network**	**Bilateral**: Anterior and posterior cingulate gyrus, pre- and post- central gyrus, precuneus, supplementary motor area, superior frontal gyrus, supramarginal gyrus, superior parietal lobule.	20 19 18	1015 232 160	28 30 57	46 77 75	60 54 53	4.93 4.04 4.13
	**Cerebellum Network**	**Bilateral**: VI, VIII a and b, IX, X, crus I	12 10	6753 3	36 27	42 39	17 8	4.8 2.4
	**Auditory Network**	**Right:** Superior and middle temporal gyrus, insula, planum temporale, planum polare, parietal operculum cortex, heschl’s Gyrus **Bilateral:** Central operculum cortex	11 10	260 180	47 72	54 39	49 40	4.54 3.64
	**Default Mode Network**	Precuneus cortex, cuneal cortex, intracalcarine cortex, supracalcarine cortex, posterior cingulate gyrus.	5 3	1816 165	49 20	34 36	36 48	4.41 4.39
	**Right Fronto-Parietal Network**	**Right:** Superior, middle, and inferior frontal gyrus, pre-central gyrus, superior parietal lobule, supramarginal gyrus, angular gyrus, lateral occipital cortex	19 18 17	1427 537 101	16 20 33	39 77 95	44 49 29	4.49 4.1 3.63
	**Left Fronto-Parietal Network**	**Left:** Superior, middle, and inferior frontal gyrus, pre-central gyrus	19 18	742 684	67 63	75 37	47 60	4.46 4.56
	**Orbitofrontal Network**	**Bilateral:** Frontal pole, frontal orbital and medial cortex, paracingulate gyrus, subcallosal cortex	5 4	2775 8	47 35	87 65	29 36	4.8 3.04
	**Dorsal Attention Network**	**Bilateral**: Lateral occipital cortex, angular gyrus, precuneus cortex, cuneal cortex, supracalcarine cortex, occipital pole, angular gyrus	3 2	4319 466	22 64	27 40	50 59	4.28 3.52
	**Basal Ganglia Network**	**Bilateral:** Thalamus, putamen, pallidum, and insular cortex, precuneus, posterior cingulate gyrus	3 2	50 20	37 38	38 37	47 40	2.75 2.53
	**Medial Temporal Network**	**Bilateral:** temporal fusiform gyrus, parahippocampal gyrus, temporal pole, inferior temporal gyrus, middle temporal gyrus, temporal occipital fusiform cortex	2 1	1388 1223	64 34	56 59	19 21	4.82 4.64
	**Ventral Stream Network**	**Right:** Superior, middle, and inferior temporal gyrus, temporooccipital part, posterior supramarginal gyrus, superior and inferior lateral occipital cortex, angular Gyrus	5 4	342 76	16 32	63 45	27 38	3.66 3.33
**ECAS Total *[Negative correlation]***								
	**Cerebellum Network**	**Bilateral**: VI, VIII a and b, IX, X, crus I	6 5 4	1112 659 166	60 28 23	35 34 27	19 17 11	5.65 4.02 3.53
	**Default Mode Network**	**Bilateral:** Precuneus cortex, cuneal cortex, intracalcarine cortex, supracalcarine cortex, posterior cingulate gyrus.	1 6 5 4	2049 51 7 6	47 65 42 47	42 23 24 89	45 49 53 27	5.17 5.39 2.95 4.99
	**Left Fronto-Parietal Network**	**Left:** Superior, middle, and inferior frontal gyrus, pre-central gyrus	19	742	67	75	47	4.46
	**Orbitofrontal Network**	**Bilateral:** Frontal pole, frontal orbital and medial cortex, paracingulate gyrus, subcallosal cortex.	3 2	4143 23	38 31	88 83	32 37	5.64 3.95
	**Basal Ganglia Network**	**Bilateral:** Thalamus, putamen, pallidum, and insular cortex	6 5	1337 467	56 42	59 53	43 26	4.55 3.99
	**Medial Temporal Network**	**Bilateral:** Temporal fusiform gyrus, parahippocampal gyrus, temporal pole, inferior and middle temporal gyrus, temporal occipital fusiform cortex	4 3	1043 113	74 30	44 60	28 21	4.92 4.23
**ECAS ALS Specific *[Negative correlation]***								
	**Orbitofrontal Network**	**Bilateral:** Frontal pole, frontal orbital and medial cortex, paracingulate gyrus, subcallosal cortex.	4 3 2 1	1500 800 200 20	32 28 21 14	80 81 72 60	31 29 40 27	5.60 3.81 2.68 2.58
	**Medial Temporal Network**	**Bilateral:** Temporal fusiform gyrus, parahippocampal gyrus, temporal pole, inferior and middle temporal gyrus, temporal occipital fusiform cortex	2 1	200 150	59 40	41 52	22 19	4.23 3.18
**ECAS ALS Non-Specific *[Negative correlation]***								
	**Default Mode Network**	**Bilateral:** Precuneus cortex, cuneal cortex, intracalcarine cortex, supracalcarine cortex, posterior cingulate gyrus.	15	2611	42	40	38	4.29
**UMN Burden *[Positive correlation]***								
	**Cerebellum Network**	**Bilateral**: VI, VII a and b, VIII a and b, Crus I, vermis VIII a	3 2 1	166 7 6	27 34 50	32 32 24	16 18 24	3.54 3.57 3.87
**Disease Progression Rate *[Positive correlation]***								
	**Sensorimotor Network**	**Right:** Middle frontal gyrus. **Bilateral**: Anterior cingulate gyrus, supplementary motor area, superior frontal gyrus, precentral gyrus. **Left:** Precuneus	6 5 4	453 272 100	45 33 29	70 68 59	61 63 65	6.01 5.22 3.99
	**Right Fronto-Parietal Network**	**Right:** Superior, middle, and inferior frontal gyrus, pre-central gyrus	6 5 4	919 192 10	28 30 22	67 92 70	61 44 63	4.81 3.85 2.89
**Symptom Duration *[Positive correlation]***								
	**Sensorimotor Network**	**Right:** Middle frontal gyrus. **Bilateral**: Anterior cingulate gyrus, Supplementary motor area, Superior frontal gyrus, Precentral gyrus.	4 3	188 12	33 59	68 46	63 68	5.28 4.11
	**Right Fronto-Parietal Network**	**Right:** Superior, middle, and inferior frontal gyrus, pre-central gyrus	1	828	28	67	61	4.61
**Left Foot Tapping *[Negative correlation]***								
	**Sensorimotor Network**	**Right:** Superior, middle, and inferior frontal gyrus, pre-central gyrus	5	13	32	63	65	2.66
**Right Foot Tapping *[Negative correlation]***								
	**Cerebellum Network**	**Bilateral**: VI, VII a and b, VIII a and b, IX, X, crus I, vermis VIII a	11 10	208 100	28 27	28 39	7 8	2.63 2.4
**Right Finger Tapping *[Negative correlation]***								
	**Basal Ganglia**	**Left: Thalamus**	4	16	40	50	20	3.90

**Abbreviations:** Intra-network resting-state functional connectivity correlation results in Amyotrophic lateral sclerosis (ALS) patients with clinical scale i.e. ALSFRS-R: ALS functional rating scale revised; ECAS: Edinburg cognitive and behavioural ALS screen; UMN; Upper motor neuron burden (FWE corrected; *p* < 0.05)

* Number of clusters represents the exact number of clusters in the fMRI analysis stayed significant after correcting the results for multiple comparisons (FWE corrected; p < 0.05).

*Cluster voxel represents number of voxels in each significant cluster.

*Peak z-stat denotes the maximum statistical value (z-stat) for the peak activity

*Coordinates were extracted from MNI 152 space

### Inter-network resting state functional connectivity

**Fig 4:**
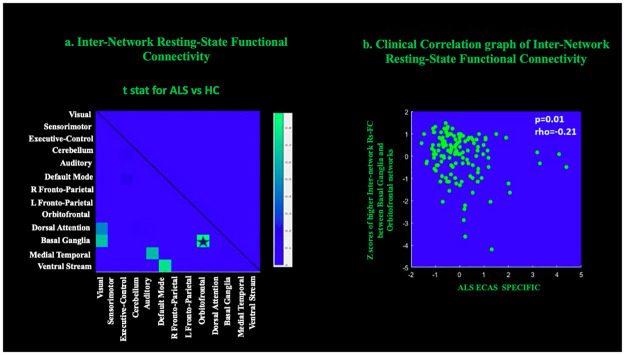
The t-test comparisons between the matrixes of partial correlation obtained from the time course series of 13 resting-state networks (RSNs) obtained from the whole group of participants. a: Higher inter-network resting-state functional connectivity (Rs-FC) between basal ganglia RSN and orbitofrontal RSN (Corrected for multiple comparisons using the family-wise error approach at p<0.05). b: Negative correlation between increased Rs-FC between basal ganglia RSN and orbitofrontal RSN and ECAS ALS Specific score (Spearmen rank correlation; [p = 0.01; r_s_ = -0.21]). Star: Higher Rs-FC in ALS patients than healthy subjects (HC).

Compared to HC, ALS patients displayed higher inter-network Rs-FC between the basal ganglia and orbitofrontal RSNs (Fig 4a) (corrected for multiple comparison approach [FWE; p < 0.05]).

### Clinical correlations

Higher inter-network Rs-FC between basal ganglia and orbitofrontal RSNs was negatively correlated with the ECAS ALS Specific score (Spearmen rank correlation; [p = 0.01; r_s_ = -0.21]) (Fig 4b). No other significant correlations were observed between inter-network Rs-FC and other clinical measures.

## Discussion

The present study aimed to investigate the role and importance of Rs-FC alterations in different networks in the pathophysiology of ALS. For this purpose, intra- and inter-network Rs-FC was examined in ALS patients and HC using an ICA technique. Compared to HC, ALS patients displayed higher intra-network Rs-FC in 11 out of 13 RSNs: sensorimotor, default mode, right and left fronto-parietal, dorsal attention, orbitofrontal, cerebellum, basal ganglia, auditory, medial temporal, and ventral stream networks. Higher intra-network Rs-FC correlated with greater disability, faster progression rate, and other clinical measures of greater disease severity (UMN scores, tapping scores, and cognitive performance). Additionally, one inter-network Rs-FC alteration was detected in ALS patients: higher functional connectivity between the orbitofrontal and basal ganglia RSNs which associated with the ECAS ALS Specific score. Collectively, these functional alterations within and between multiple RSNs are useful in characterizing and further understanding the pathophysiology of ALS. Notably, the CALSNIC cohort provides the opportunity to characterize effects over participants enrolled from multiple sites using a harmonized MRI and clinical protocol.

### Intra-network functional connectivity

#### Sensorimotor RSN

ALS patients displayed higher intra-network Rs-FC in the bilateral sensorimotor RSN involving the superior, middle, and inferior frontal gyrus, anterior and posterior cingulate gyrus, pre- and post-central gyrus, supramarginal gyrus, precuneus, supplementary motor area and parietal lobes. These areas play a key role in integrating sensory and motor information [50–52], particularly for processing sensory information into action [53] or for executing motor tasks and/or sensorimotor components [10, 11]. In ALS, functional changes in the sensorimotor RSN have been consistently reported [7, 12–15, 17–19]. Few such studies have reported enhanced Rs-FC within the sensorimotor RSN using an ICA approach [17, 18] whereas others have used an *a-priori* approach [12, 22] suggesting an underlying mechanism of neurodegeneration in the motor cortex of ALS patients. Enhanced Rs-FC in ALS patients [22] were also reported using network analysis indicating focal reorganisation and remodulation of functional brain networks. Our finding of a Rs-FC increase in the sensorimotor RSN provides further evidence for a breakdown of neuronal circuits in ALS patients [54]. In addition, the present study found that enhanced Rs-FC within the sensorimotor RSN correlated with faster disease progression rate and longer symptom duration. This is in alignment with previous findings [7, 13] suggesting an association between a dynamic pattern of functional alterations in the sensorimotor RSN of ALS patients and an increase in motor impairment [7, 55]. The present study also found a correlation between higher intra-network Rs-FC in the sensorimotor RSN and lower ALSFRS-R. The findings are supportive of an underlying mechanism of increased neuronal connectivity in the motor cortices of ALS patients, potentially acting as a predictor of motor decline and higher clinical disability [56]. Moreover, higher intra-network Rs-FC in the right hemisphere of the sensorimotor RSN (superior, middle, inferior frontal gyrus, and pre-central gyrus) significantly correlated with the left foot tapping scores. Tapping is related to UMN functioning [57] which is thought to be affected by degeneration of the primary motor cortex in ALS [54].

In addition, the present study found that enhanced Rs-FC within the sensorimotor RSN correlated with faster disease progression rate and longer symptom duration. This is in alignment with previous findings [7, 13] suggesting an association between a dynamic pattern of functional alterations in the sensorimotor RSN of ALS patients and an increase in motor impairment [7, 55]. The present study also found a correlation between higher intra-network Rs-FC in the sensorimotor RSN and lower ALSFRS-R. The findings are supportive of an underlying mechanism of increased neuronal connectivity in the motor cortices of ALS patients, potentially acting as a predictor of motor decline and higher clinical disability [56]. Moreover, higher intra-network Rs-FC in the right hemisphere of the sensorimotor RSN (superior, middle, inferior frontal gyrus, and pre-central gyrus) significantly correlated with the left foot tapping scores. Tapping is related to UMN functioning [57] which is thought to be affected by degeneration of the primary motor cortex in ALS [54]. Surprisingly, other studies have reported decreased BOLD signals in the sensorimotor RSN using an ICA approach [19]. The contradictory findings between the former and present study are likely due to the differences in patient characteristics and data acquisition parameters. Additionally, previous studies have different analytical methodologies which may also have likely triggered the differences in the findings.

#### Default mode RSN

ALS patients displayed higher intra-network Rs-FC in the posterior part of the default mode RSN involving the bilateral precuneus cortex, cuneal cortex, supra- and infra- calcarine cortex, posterior cingulate gyrus, angular gyrus, superior and inferior lateral occipital cortex, supramarginal gyrus, and middle temporal gyrus. The default mode RSN is mainly involved in the regulation of complex cognitive and social functions [58–61]. More specifically, the anterior default mode RSN is involved in self-referential mental thoughts [62] and the posterior default mode RSN is mainly involved in directed attention, such as day-dreaming and scene-construction [62]. In ALS patients, several studies have examined the default mode RSN using ICA [13, 17–20] and seed-based approaches [7, 9]. For example, compared to HC, Mohammadi et al (2009) reported decreased Rs-FC in the frontal and temporal areas [18] whereas Agosta et al (2013) reported increased Rs-FC in the left precuneus and decreased Rs-FC in the right inferior orbitofrontal gyrus [13]. When compared to patients with FTD, ALS patients were found to have decreased Rs-FC in the posterior cingulate cortex [20]. This mixed picture of functional response within the default mode RSN may suggest high variability in clinical and cognitive features [13, 17–20, 63]. Because this network is known to be more active when the mind is free to wander and not engaged in any task-driven activity [64], the present findings may indicate that individuals with ALS synchronize more extra-motor regions (i.e. temporo-occipital areas) than HC in the posterior default mode RSN. Supporting this argument, atypical patterns of cerebral degeneration have previously been observed in the temporo-occipital brain regions of ALS patients [65–68]. Increased cortical functional connectivity within the posterior cingulate gyrus and occipital brain regions has also been recorded by resting-state magnetoencephalography (MEG) [69]. Higher Rs-FC is suggestive of higher functional activity at rest [70, 71], in turn reflecting the clinical aspects of cognitive and behavioral impairment in ALS.

In addition, higher intra-network Rs-FC in the posterior cingulate gyrus and precuneus of the default mode RSN correlated with lower ALSFRS-R and cognitive tests (ECAS Total and ECAS ALS Non-specific). The ECAS Total score reflects overall cognitive status and ECAS ALS Non-specific captures memory and visuospatial dysfunction [72]. Both the posterior cingulate gyrus and the precuneus have multiple functional roles, and thus damage to these regions results in various cognitive, emotional, and behavioral disturbances [73, 74] and especially executive dysfunction [72]. Since these extra-motor features are more frequently reported in ALS [4, 75], the observed correlation in the present study confirm increased clinical disability beyond muscle weakness i.e. decline of certain cognitive functions, impaired social cognition, and changes in the perception and processing of emotions in ALS patients. This also aligns with the overall characteristics of ALS that as the disease progresses, brain regions beyond those involved in motor control, that are more involved in cognitive processing, become affected and contribute to worsening clinical symptoms [68, 73].

#### Dorsal attention RSN

Another interesting finding of the present study is that intra-network Rs-FC is higher in the dorsal attention RSN of ALS patients. The dorsal attention RSN, also known as "task-positive RSN" is most active during a stimulus-based task and is typically anti-correlated with engagement of the default mode RSN [64, 76, 77]. Notably, no other ALS neuroimaging studies to date have observed activation of the dorsal attention RSN. However, brain regions within the dorsal attention RSN have been recorded individually in the context of higher-level cognitive processing several task-based fMRI, MEG, and electroencephalography studies of ALS [69, 73, 78, 79]. These studies shed light on functional disturbances in the extra-motor brain networks reflective of cognitive changes in the cortical structure of the cerebral hemisphere. In the present Rs-FMRI study, the dorsal attention RSN was found mainly to encompass bilateral parieto-occipital brain areas such as the precuneus, lateral occipital cortex, angular gyrus, cuneal cortex, and supracalcarine cortex. These brain regions mainly subserve cognitive domains such as attention, executive functions, visuospatial functions, episodic memory, language, and number processing [80–83]. The increased Rs-FC in the dorsal attention RSN could be a compensatory attempt [56] to preserve these cognitive functions and thus maintain attentional resources in the presence of cognitive fatigue [84, 85].

Higher intra-network Rs-FC in the dorsal attention RSN was also found to be negatively correlated with the ALSFRS-R. This supports the idea that cognitive impairment, here specifically subserved by abnormal Rs-FC of the dorsal attention RSN, contributes to the functional impairment in addition to that due to motor weakness.

#### Right and left fronto-parietal RSN

ALS patients also showed enhanced intra-network Rs-FC mostly in the frontal and parietal brain regions of the right and left fronto-parietal RSN. The involvement of this RSN in ALS pathology has been addressed twice using ICA methods [13, 18]. One study observed increased Rs-FC in the left fronto-parietal RSN of ALS patients correlating with clinical and cognitive deficits, suggesting that network dysfunction between the frontal and parietal brain regions could induce subtle changes in executive functions [13]. The other study did not detect significant group differences [18]. Thus, at present there is no consensus on involvement of the fronto-parietal RSN in ALS pathology. The fronto-parietal RSN tends to consolidate neuronal signals from multiple brain regions and/or diverse brain RSNs, acting as a relay center for executing complex cognitive functions [5, 76, 86–88]. In the present study, enhanced intra-network Rs-FC in the fronto-parietal RSN could be a dysfunctional outcome of the frontal and parietal brain regions [13] reflecting the inability to maintain the inhibitory/excitatory balance [89] and/or proper functional organization [13]. These effects may potentially impact the complex cognitive circuitry involved in processing higher cognitive and executive functions [90–94].

A negative correlation was also observed between intra-network Rs-FC in the right and left fronto-parietal RSN, and ALSFRS-R score, indicating Rs-FC changes of brain networks involving connections to the frontal and parietal brain regions is due to the progression of clinical dysfunction in ALS. This anti-correlation confirm neurodegeneration in ALS and is commonly reported as the insufficient response of clinical performances in fronto-parietal RSN of ALS leads to attention lapse, poor cognitive task performance, and improper well-being of mental presentation [95]. Moreover, positive correlations with disease progression rate and symptom duration suggest higher neuronal synchronization is linearly associated with an average disease progression rate and severity of clinical symptoms [96, 97]. Last, Rs-FC in the left fronto-parietal RSN was associated with cognitive performance (ECAS Total) indicating that the underlying neural mechanism might be analogous to the dysfunctions of frontal and parietal brain regions [79, 98–101]. Overall, the present findings suggest that functional connectivity in the fronto-parietal network is crucial for instantiating and flexibly modulating cognitive control [102].

#### Cerebellum RSN

ALS patients showed higher intra-network Rs-FC in the cerebellum RSN (left I-IV, right Crus II, and bilateral VI, VII a and b, IX, X, Crus I, Vermis VIII a). These areas regulate numerous motor and extra-motor functions: lobules I-IV and IX regulate tapping and sensorimotor functions; lobule X regulates balance, posture, reflexes, and eye movements; lobule IV, VII, Crus I and II are implicated in higher cognitive functions such as language, working memory, and visuomotor processes [103]. The functional importance of these cerebellar lobules has been reported previously in ALS, associated with motor and cognitive decline [8, 12, 104]. Several studies have reported Rs-FC changes in the cerebellum using a seed-based approach, suggesting a functional response to the disease-related mechanism [8, 12, 105]. Using ICA, another study did not detect any significant Rs-FC differences within the cerebellum RSN [17]. As the cerebellum is involved in regulating both motor and cognitive functions [103, 106–108], the present findings of higher intra-network Rs-FC in the cerebellar lobules potentially reflects the generalized spread of cerebellar functional alterations as part of compensating for motor and cognitive decline [109]. Supporting this argument, higher cerebellar Rs-FC was found to be negatively correlated with ALSFRS-R scores, and with poor cognitive and tapping performance, suggesting that increasing disability is associated with widespread cerebellar dysfunction [105]. Furthermore, a positive association was found between higher intra-network Rs-FC and higher UMN burden scores. Tapping assesses UMN function [110] and provides useful measure of UMN degeneration in the motor cortex [54, 57]. Lower tapping scores indicate worse motor performances and are suggestive of greater UMN degeneration and functional loss [57, 111]. In line with the role of the cerebellum in facilitating highly practiced movement [112], the present findings link well with dysfunctions in balance, posture, coordination, speech, and other smooth and balanced muscular activities in ALS patients.

#### Basal ganglia RSN

Higher intra-network Rs-FC was found bilaterally in the basal ganglia RSN including the thalamus, putamen, pallidum, insula, and precuneus cortex of ALS patients. Alterations of the whole basal ganglia RSN have not been reported previously in the ALS literature. However, the constituent brain regions have been reported very often in the context of motor and extra-motor ALS manifestations [12, 113], suggesting the major role and importance of the basal ganglia and the associated cortico-basal ganglia-thalamo-cortical loops in the pathophysiology of the disease [113–115]. Basal ganglia degeneration has been touted as a possible imaging biomarker of ALS [113]. The structural and functional relevance of the basal ganglia have also been discussed with respect to widespread dysfunctional connectivity, volumetric atrophy, cortical thinning, and white matter degeneration in ALS [22, 55, 113, 114, 116–118]. While the basal ganglia and its associated pathways are classically known in ALS pathology for preserving the intact motor and cognitive abilities [113, 114], our findings can be interpreted as alterations in the fronto-striatal circuitry is implicated in the dysfunction of motor outcomes, and impaired cognitive, and behavioural flexibility in ALS patients [119–121].

Furthermore, increased Rs-FC of the basal ganglia RSN correlated with lower clinical scores of ALSFRS-R, ECAS Total, and right finger tapping. These correlations align with the overall characteristics of basal ganglia pathology in ALS. The basal ganglia, through their afferent and efferent pathways, not only influence the primary motor cortex but also premotor and prefrontal cortices that are involved in language and cognitive functions [119–121]. Therefore, increasing dysfunction of basal ganglia RSN and its associated pathways is likely contributing to the worsening of clinical symptoms, including greater disability and motor and cognitive decline.

#### Auditory, medial temporal, and ventral stream RSNs

Higher intra-network Rs-FC in the auditory, medial temporal, and ventral stream RSNs was found for ALS patients. These have not previously been implicated in ALS. ALS pathology in the cerebrum is thought to develop first in the motor regions with progressive spread in frontotemporal regions (with the last pathological stage including the medial of the temporal lobe) [9, 122, 123]. A recent Rs-FMRI study has discussed the importance of temporo-occipital brain regions in the pathophysiology of ALS, suggesting their involvement at the later stages of disease [66]. In the present study, therefore, higher intra-network Rs-FC within the auditory, medial temporal, and ventral stream RSNs may suggest a compensatory recruitment of additional resources to overcome early stage dysfunction [124].

Moreover, higher intra-network Rs-FC in the auditory, medial temporal, and ventral stream RSNs negatively correlated with ALSFRS-R, indicating that physical impairment is associated with the altered neural connectivity accompanying the progression of loss in functional outcomes [105, 125]. Higher intra-network Rs-FC in the medial temporal RSN also correlated negatively with ECAS Total and ECAS ALS Specific scores, indicating that greater medial temporal connectivity is associated with worse cognitive performance specifically involving language, verbal fluency, and executive functions [95, 98, 126]. The abnormal Rs-FC involving the mesial temporal lobe is in line with frontotemporal lobar degeneration. Because the significance of hyper-connectivity is poorly understood in ALS in the context of cognition, these correlation results shed additional light on the medial temporal involvement and the nature of cognitive decline in ALS patients consistent with the characteristics of later-stage disease pathology.

#### Orbitofrontal RSN

ALS patients had higher intra-network Rs-FC bilaterally in the frontal pole, frontal orbital, frontal medial, paracingulate gyrus, and subcallosal cortex of the orbitofrontal RSN. This particular RSN occupies the prefrontal cortex of the frontal lobe [127] and is involved in the processing of memory, executive functions, reward, decision making, goal-directed behavior, and control of inhibitory functions [128–137]. Regions of the orbitofrontal RSN are also engaged in processing emotional responses and social behavior such as empathy, external environmental stimuli, predicting future events, and thoughts about the self and others [138]. The orbitofrontal RSN has been previously discussed in ALS in the context of stimulus-driven tasks [139] given the importance of these networked regions in cognition and in executive processing. Using graph-theory methods, enhanced resting-state fluctuations in the right orbital frontal and prefrontal cortex were proposed as a means to assess disease severity in the later stages of ALS [63]. A decreased functional response in the right inferior orbitofrontal gyrus of the default mode RSN has also been reported to suggest cognitive impairment [13].

As discussed above, motor-related Rs-FC changes in the motor areas of the frontal cortex are pronounced at stage one of ALS pathology. Extra-motor regions including the prefrontal cortex and orbitofrontal cortex occur at stage three [54, 122, 140]. This suggests that the findings in the present study of ALS Rs-FC changes within the orbitofrontal RSN reflect dysfunction in the extra-motor system caused by progressive neurodegeneration at the later stages of the disease [63, 122] as also observed in other disorders such as Parkinson’s disease gait [141–144]. From the literature already discussed, it is speculated that the higher intra-network Rs-FC is a compensatory mechanism [56] within the orbitofrontal RSN to maintain cognitive, affective, and executive functions. This notion is further supported by the negative correlation of higher orbitofrontal Rs-FC with lower ALSFRS-R and the negative correlations with ECAS Total and ECAS ALS Specific scores.

### Inter-network resting-state functional connectivity

#### Basal ganglia and orbitofrontal RSNs

ALS patients displayed higher inter-network Rs-FC between the basal ganglia RSN and the orbitofrontal RSN. The inter-functional relationship between these RSNs has not been assessed previously in the ALS literature, but the structural and functional relevance of basal ganglia and orbitofrontal brain regions in ALS pathology have been studied extensively as described above, indicating that the disease can produce widespread neurodegeneration [13, 63, 113, 115]. Studies have reported involvement of basal ganglia grey matter pathology in terms of the degree of volumetric changes, shape, and density in ALS [113, 115] suggesting an association of the basal ganglia with widespread areas of the neocortex [115] and/or more specific dysregulation of the fronto-striatal network [113]. Moreover, dysfunction in the projections of the basal ganglia and orbitofrontal cortex may lead to altered fronto-striatial connectivity patterns [145]. Cortico-basal ganglia circuits are involved in monitoring the motor and cognitive aspects of altered cortical processes [145–147] and thus influence sensorimotor, limbic and cognitive functions [121, 148, 149]. These circuits are known to play a key role in ALS pathology [150] and act as an underlying substrate in the control of motor and cognitive capabilities of the prefrontal cortex [149]. The orbitofrontal cortex also has reciprocal anatomical connections with the basal ganglia controlling multiple functions, such as decision-making [151, 152].

Based on this literature, the present findings of increased Rs-FC between the basal ganglia RSN and orbitofrontal RSN likely are a consequence of compensatory processes attempting to preserve motor and cognitive functions in the presence of ALS [56]. Furthermore, a negative correlation of higher Rs-FC of these RSNS with lower ECAS ALS Specific score is consistent with the idea that damage to the fronto-striatal circuits (as can occur in ALS) may lead to the impaired cognitive and executive functions [145, 147]. Overall, the present findings suggest that ALS pathology significantly alters the interplay between the basal ganglia and orbitofrontal RSNs.

## Conclusion

This study highlights the role and importance of multiple RSNs in the pathophysiology of ALS, and sheds light on functional disturbances within and between RSNs. Both motor and extra-motor RSNs were found to be altered in ALS, and Rs-FC changes were associated with clinical measures of motor, cognitive, and general disease status. ALS is a multi-system disorder, confirmed by the extent of diffuse involvement of RSNs [5].

Previous neuroimaging literature has demonstrated increased Rs-FC in ALS patients [17, 69, 105] this methodology holding promise as a neuroimaging biomarker [143, 153]. The present study findings are supportive of prior works that higher Rs-FC represents a pathobiological feature related to key clinical aspects of the disease [71, 89, 154]. Higher inter-network Rs-FC could also indicate an early compensatory or adaptive response mechanism to overcome motor and cognitive decline, mediated by functional reorganization and plasticity-related changes [56]. The association between higher neuronal synchronization and disease-related modulatory response in ALS is also suggestive of such changes [155].

From a methodological perspective, the present work also highlights the utility of the ICA approach to explore whole brain RSN functional connectivity in ALS. The multi-center CALSNIC Rs-FMRI data facilitated exploration of RSNs in a large population sample in unprecedented detail, revealing the involvement of specific networks in ALS not previously described. Discrepancies with previous literature could be due to sample size and/or methodological differences, as well as patient variability and recruitment bias.

## Limitations

There are limitations to our study. First, the multicenter approach introduces site-dependent bias and variability in clinical and imaging data. However, a harmonized imaging and clinical protocol was adopted across all sites and the Rs-FMRI data were carefully processed in a stringent and standardized manner to extract Rs-FC information. Before performing the statistical analysis, the data were carefully de-noised to suppress confounding effects including head motion [39] followed by additional motion parameter analysis to remove bias from motion artifact [43]. The statistical analysis also included a multiple linear regression model accounting for multiple co-variates of no interest (age, sex, motion parameters, site, etc.) to increase the likelihood that the observed Rs-FC changes related to disease state (ALS, HC) and clinical variables.

Second, the association of network connectivity with clinical disease staging was not explored. This could be explored in the future by subgrouping patients according to the King’s or Mitos staging systems.

Third, associations between functional and structural changes were not explored. Such association are important to investigate, to assess the extent that the observed increases in Rs-FC reflect grey matter or white matter pathology [14]. An association between functional alterations in large-scale RSNs and structural damage could be addressed accordingly in future studies.
